# Chitosan-Based Biomaterials for Tissue Regeneration

**DOI:** 10.3390/pharmaceutics15030807

**Published:** 2023-03-01

**Authors:** Yevgeniy Kim, Zharylkasyn Zharkinbekov, Kamila Raziyeva, Laura Tabyldiyeva, Kamila Berikova, Dias Zhumagul, Kamila Temirkhanova, Arman Saparov

**Affiliations:** Department of Medicine, School of Medicine, Nazarbayev University, Nur-Sultan 010000, Kazakhstan

**Keywords:** chitosan biomaterials, functionalized polysaccharides, tissue regeneration, tissue engineering, drug delivery, bone regeneration, cartilage regeneration, dental regeneration, cardiac regeneration, nervous regeneration

## Abstract

Chitosan is a chitin-derived biopolymer that has shown great potential for tissue regeneration and controlled drug delivery. It has numerous qualities that make it attractive for biomedical applications such as biocompatibility, low toxicity, broad-spectrum antimicrobial activity, and many others. Importantly, chitosan can be fabricated into a variety of structures including nanoparticles, scaffolds, hydrogels, and membranes, which can be tailored to deliver a desirable outcome. Composite chitosan-based biomaterials have been demonstrated to stimulate in vivo regeneration and the repair of various tissues and organs, including but not limited to, bone, cartilage, dental, skin, nerve, cardiac, and other tissues. Specifically, de novo tissue formation, resident stem cell differentiation, and extracellular matrix reconstruction were observed in multiple preclinical models of different tissue injuries upon treatment with chitosan-based formulations. Moreover, chitosan structures have been proven to be efficient carriers for medications, genes, and bioactive compounds since they can maintain the sustained release of these therapeutics. In this review, we discuss the most recently published applications of chitosan-based biomaterials for different tissue and organ regeneration as well as the delivery of various therapeutics.

## 1. Introduction

Chitosan is a polysaccharide that is widely used for various biomedical applications. It is obtained through partial deacetylation of chitin, which is the second most abundant polysaccharide and is found in the exoskeletons of crustaceans and insects, and in the cell walls of fungi [1]. Depending on which agents are used to extract chitin from living organisms and transform it into chitosan, there are two methods of chitosan synthesis, namely chemical or biological [2]. Chemical methods rely exclusively on chemicals, whereas biological techniques utilize either enzymes or bacteria for chitosan production. Both techniques commonly involve three steps, which are demineralization, deproteinization, and deacetylation. The first step is aimed at removing CaCO_3_, which in a chemical approach, is performed using hydrochloric acid. In the following step, proteins and other organic compounds are eliminated with an alkaline solution generating chitin. The third step of synthesis involves the deacetylation of chitin commonly using sodium hydroxide solution. On the other hand, in the biological approach of chitosan preparation, these three steps are performed by bacteria and/or enzymes. Chemical methods of chitosan synthesis tend to be cheaper, more rapid, less complicated and produce a higher yield with greater degrees of deacetylation compared to biological alternatives. Nevertheless, biological methods could generate chitosan with superior mechanical properties.

Chitosan has a number of advantageous properties such as biocompatibility, biodegradability, and antimicrobial activity among others [3]. These characteristics make chitosan a very attractive biomaterial to use in a number of biomedical applications including tissue engineering, drug delivery, vaccine administration, and medical device production [4]. Furthermore, chitosan can be fabricated into a variety of formulations such as nanoparticles, nanofibers, scaffolds, hydrogels, membranes, films, and many others which makes it suitable for particular biomedical applications [5]. A variety of techniques are utilized to prepare different chitosan formulations such as ionic gelation, solvent evaporation, reverse micellar, cross-linking, self-assembling, sieving, spray drying, and freeze-drying [6]. For instance, ionic gelation and sieving methods are used to produce chitosan nanoparticles while physical and chemical cross-linking are deployed to synthesize hydrogels [7]. It is important to mention that for biomedical applications, chitosan tends to perform as well as other marine-derived biopolymers such as alginate, ulvan, carrageenan, and others in terms of safety, biocompatibility, and bioactive properties such as interactions with cells and biomolecules [8]. Nonetheless, compared to many other biomaterials of marine origin, chitosan has an important limitation—it is poorly soluble in alkaline and neutral aqueous solutions [7,8]. Other limitations of chitosan include burst release of drugs in certain tissues (e.g., stomach), inadequate mechanical properties, difficulty to control pore size during synthesis, and others [7,9]. However, there are a number of strategies to overcome the limitations of pure chitosan including chemical modifications and combination with other natural or synthetic biopolymers.

Chitosan can be chemically and physically modified to address its limitations and design formulations tailored to deliver a desirable outcome [10]. Thus, chitosan possesses hydroxy- and amino-functional groups, which can be subjected to carboxyalkylation, sulphonation, acetylation with acids and esters, and with the addition of sugars, producing derivatives with improved solubility in water, enhanced regenerative properties, and ameliorated drug-delivery capabilities [11,12]. Moreover, the addition of other chemical groups enriches the bioactive properties of chitosan, enabling it to interact with a greater range of active molecules and cells. Another approach to address the inadequacies of pure chitosan and enhance its properties is to combine it with different natural and synthetic polymers. In particular, chitosan is frequently used in combination with various natural biomaterials, including but not limited to, gelatin, hyaluronic acid, alginate, collagen, silk fibroin, and other compounds [1]. In addition, composites of chitosan and various synthetic polymers such as polyvinyl alcohol, polypyrrole, and poly(-caprolactone), polyvinylpyrrolidone among others are widely utilized. These synthetic compounds were shown to greatly ameliorate the native properties of chitosan making it a more efficient drug delivery agent and improving its regenerative potential.

One promising biomedical application of chitosan-based biomaterials is for the sustained delivery of therapeutics. In particular, chitosan-based delivery systems have been developed for a range of drugs, genes, and biomolecules, and can be administered via a variety of routes, including oral, parenteral, topical, buccal, and sublingual [13,14,15]. Chitosan is a good candidate for controlled drug-delivery systems due to its exceptional mucoadhesive properties, which allow it to bind to glycosaminoglycans and proteoglycans of the mucus membranes [16]. In addition, chitosan can significantly enhance permeability through epithelial tight junctions which enables efficient transepithelial drug delivery [17]. Chitosan-based drug-delivery systems have been explored for use in the treatment of various conditions including cancer, Crohn’s colitis, Alzheimer’s, and Parkinson’s diseases [18,19,20,21]. It has been shown to improve permeation and bioavailability as well as mediate the sustained release of medications such as antibiotics, antidiabetic drugs, and anticancer treatment [10,16]. Aside from medications, chitosan biomaterials have been utilized to deliver cytokines, bioactive trace elements, nucleic acids, and vaccines [1,3,5,22].

**Figure 1:**
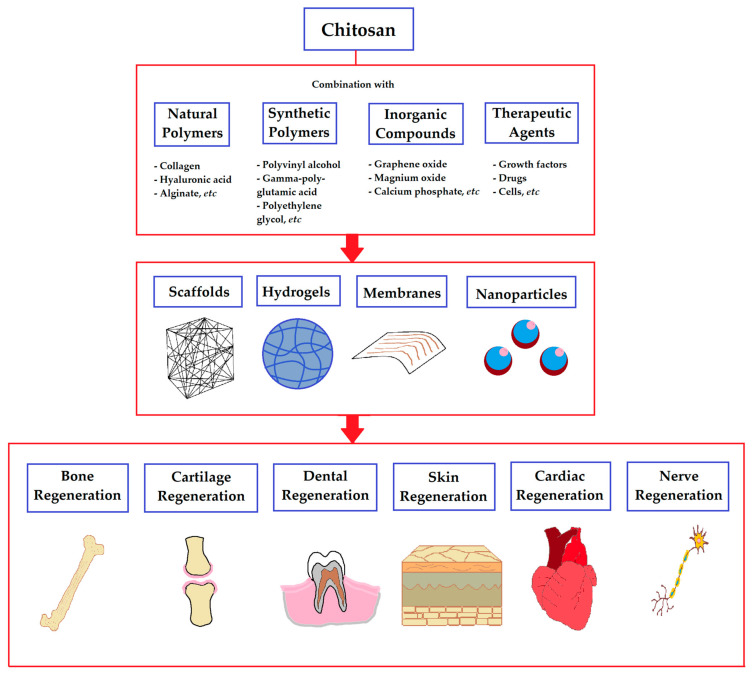
Use of chitosan biomaterials for tissue regeneration. Chitosan can be formulated alone or in combination with natural and synthetic polymers, inorganic compounds, and therapeutic agents into a variety of structures including scaffolds, hydrogels, membranes, and nanoparticles. These biomaterials have been demonstrated to promote regeneration and repair of bone, cartilage, dental, skin, cardiac, and nerve tissues.

Another important therapeutic application of chitosan-based biomaterials is in regenerative medicine due to their ability to stimulate damaged tissue repair and new tissue formation [23]. Indeed, numerous studies have utilized chitosan formulations to regenerate bone, dental, cartilage, corneal, skin, nervous, cardiac, and other tissues (Figure 1) [24,25]. For instance, chitosan biomaterials were shown to induce mineralization, osteogenesis, and repair of bone defects in different animal models of bone injuries [12,26]. Similarly, chitosan formulations can support the growth and differentiation of chondrocytes, promote chondrogenesis, stimulate the secretion of cartilage-specific proteins, and mediate the formation of novel cartilage tissue [5,27]. Chitosan-based biomaterials were also demonstrated to have several positive effects on heart and blood vessel regeneration. Specifically, chitosan-containing hydrogels were effective at preventing adverse cardiac remodeling and improving cardiac function in the experimental models of cardiomyopathy and myocardial infarction [28,29]. Moreover, certain composite chitosan formulations efficiently support electrical conduction, which is crucial for the regeneration of myocardium [30]. Chitosan also has great potential for skin regeneration and wound healing owing to its hemostatic and antimicrobial properties [5,10]. Multiple studies have demonstrated that chitosan biomaterials could facilitate cell proliferation at the wound site and mediate complete wound regeneration and epithelial reconstruction [5,31].

In this review, we will discuss the latest applications of chitosan-based biomaterials for tissue regeneration and repair. Specifically, we will focus on the use of chitosan biomaterials for bone, cartilage, dental, skin, cardiac, and nervous tissue.

## 2. Bone, Cartilage, and Dental Tissues

### 2.1. Bone Regeneration Using Chitosan-Based Biomaterials

Different types of chitosan-based biomaterials such as dried scaffolds, injectable hydrogels, nanofiber membranes, and others have been utilized for bone regeneration [26,32,33]. Dried chitosan scaffolds are generally combined with other natural or synthetic polymers since in its pure form, they have low bioactivity and mechanical stability. Such composite chitosan scaffolds have been shown to efficiently promote endogenous bone repair [34]. The dried scaffolds can be fabricated by freezing and lyophilization, salt leaching, and electrospinning [26]. Injectable hydrogels are another type of chitosan scaffold, but in contrast to dried scaffolds, they are prepared in liquid form. The benefits of injectable hydrogels over other chitosan biomaterials for bone regeneration include their minimal invasiveness, high water content, ability to fill bone defects of various irregular shapes, and several others [12]. Nanofiber membranes, which are prepared via an electrospinning method, are yet another formulation of chitosan that hold great potential for guided bone regeneration due to their ability to induce mineralization, osteogenic differentiation, and angiogenesis [35,36,37]. Importantly, all three of the aforementioned chitosan-based biomaterials can be used to deliver bioactive molecules and cells to enhance the regeneration of the osseous tissue. Thus, several studies have deployed chitosan-based scaffolds, injectable hydrogels, or nanofibrous membranes to deliver bone minerals, various growth factors and cytokines, as well as stem cells of various origins and other therapeutic agents that improved bone regeneration [7,26,36,38].

Scaffolds are one of the most popular types of chitosan formulations used for bone regeneration. Several research groups have applied such biomaterials to deliver bioactive molecules and microelements to the osseous tissue to promote new bone formation. In a study by Huang and colleagues [39], a composite scaffold made of magnesium oxide nanoparticle-coated eggshell particles and chitosan was synthesized to deliver magnesium ions and bone morphogenic protein 2 (BMP2) to stimulate osteogenesis in vitro and in a rat calvarial bone defect model. It was found that the scaffold could activate osteogenic differentiation of human adipose-derived stem cells, which was evidenced by the increased expression of osteogenesis-related proteins such as alkaline phosphatase, collagen, sialoprotein, osteocalcin, and osteopontin. The authors reported the mechanism by which this happened, namely, by the synergistic activation of Akt and ERK1/2 phosphorylation by BMP2 and magnesium ions. The chitosan-based delivery system also positively affected the repair of bone injuries in the rat model. Specifically, after treatment with the scaffold, the bone defects were completely filled with the new osseous tissue. The scaffold-induced osteogenesis was also confirmed by increased bone density observed on micro-computed tomography and fluorescence quantitative analysis. Importantly, van Gieson’s picrofuchsin staining revealed that the newly formed bone had a woven trabecular structure. Aside from bioactive molecules and microelements, chitosan scaffolds can also be used for the delivery of stem cells. Bakopoulou and colleagues demonstrated that biomimetic chitosan-gelatin scaffolds could support the viability and differentiation of dental pulp stem cells in culture and in immunocompromised mice [40]. Thus, the cells remained viable and were expressing osteo- and odontogenic differentiation markers inside the scaffolds. Moreover, there was calcium phosphate-rich tissue produced within the scaffold by the cells. The formation of novel osseous tissue was also observed in vivo in the cell-free scaffold, scaffold with the dental pulp stem cells, and scaffold loaded with dental pulp stem cells pretreated with recombinant human BMP-2. In particular, histology confirmed the formation of the fully mineralized bone with osteocytes 6–10 weeks after the treatment. Importantly, the greatest amount of new tissue was found in the case of the scaffold with BMP-2-pre-treated cells. Georgopoulou and colleagues also utilized chitosan-gelatin scaffolds as a delivery system for stem cells for bone regeneration. Specifically, they demonstrated that the scaffolds could support the adhesion, survival, and proliferation of pre-osteoblastic cells and bone marrow mesenchymal stem cells (BM-MSCs) [41]. In addition, the scaffolds promoted the osteogenic differentiation of the BM-MSCs, which was evidenced by the increased expression of the RUNX2, ALP, and OSC markers. Furthermore, the chitosan-gelatin biomaterial promoted the formation of a new extracellular matrix (ECM) in a mouse femur orthotopic model of implantation.

Injectable hydrogels containing chitosan have been also demonstrated to enhance bone tissue regeneration. Specifically, there are a number of publications reporting the use of such biomaterials for the incorporation of osteoinductive compounds, bioactive molecules, and medications. For instance, recently, Lee and colleagues have utilized glycol chitosan and oxidized hyaluronic acid injectable hydrogel to optimize the delivery of graphene oxide, a known osteoinductive material [42]. It was shown that the hydrogel could be used to transfer required quantities of graphene oxide to promote osteogenesis in cell culture and in a rat model of calvarial defects. Thus, the graphene-oxide loaded hydrogel induced osteogenic differentiation of human adipose-derived mesenchymal stem cells, which was confirmed by positive staining for alkaline phosphatase activity and calcium deposition as well as the increased expression of osteogenic markers COL1, OCN, BSP, and RUNX2. Furthermore, the treatment mediated the complete closure of the bone defect in the rat model four weeks after the injection. In vivo bone regeneration after treatment was also demonstrated by micro-computed tomography and Masson trichrome staining. It is important to note that the graphene oxide incorporated hydrogel appeared significantly superior to the hydrogel alone both when applied in cell culture and in the animal model. Chitosan-based injectable hydrogels have also been deployed to deliver medications. Jin Tao and colleagues [43], for instance, fabricated a thermosensitive chitosan-glycerol phosphate hydrogel/nanoparticle system to carry vancomycin to the infected bone and to enhance its repair. The researchers demonstrated that the drug-delivery system mediated a sustained release of vancomycin for 26 days, significantly reduced the number of *S. aureus* colonies after 15 days, and eliminated the bacteria from infected osteoblasts after 2 days of in vitro treatment. In addition, the carrier system was efficient in an animal model of chronic osteomyelitis. Thus, 8 weeks following hydrogel implantation, bone inflammation was significantly reduced while the bone repair was enhanced, which was demonstrated by a reduction in inflammation markers as well as radiographic and histopathological examination. A similar thermosensitive injectable hydrogel drug-delivery system made of chitosan and glycerol phosphate was engineered by Petit and colleagues [44]. The carrier system was intended to transfer statins that are known to increase osteogenesis and decrease bone resorption. In particular, nanoemulsions of atorvastatin or lovastatin were incorporated into the hydrogel. The therapeutic systems efficiently promoted novel bone formation in rat bone of calvarial defect 15 days after injection, which was shown by the alizarin red staining for calcium and immunofluorescence staining of calcium incorporation. It should also be mentioned that the release of the drugs from the nanoemulsions in chitosan hydrogels in vitro followed a linear pattern peaking at 18–20 h. On the other hand, chitosan-based injectable hydrogels can act not only as mere delivery systems for various therapeutic compounds but can also promote bone repair on their own. In a study by Cui and colleagues [45], a highly osteoinductive injectable in situ forming hydrogel consisting of methacrylated glycol chitosan and montmorillonite was fabricated. The hydrogel alone was able to recruit native cells and mediate osseous tissue regeneration in a calvarial defect model in mice even without the delivery of stem cells, bioactive molecules, or other therapeutic agents. In particular, six weeks after the injection, the new osteoid bone was observed on the Masson trichrome staining, which was also evidenced by micro-computed tomography and positive immunohistochemical staining for RUNX2 and OCN. Interestingly, when the hydrogel was made only of methacrylated glycol chitosan, it could not induce the same regenerative effects as hydrogel consisting of methacrylated glycol chitosan with montmorillonite. Thus, composite injectable hydrogels showed great potential for bone regeneration when used alone or as a delivery system for drugs and bioactive compounds.

The application of nanofiber membranes is another approach of chitosan fabrication for bone regeneration. Multiple in vitro studies have demonstrated that chitosan-based composite membranes could support the bioactivity of osteoblasts and induce mineralization [46,47,48]. Importantly, such nanofiber membranes can also mediate the controlled and sustained release of various bioactive compounds. Thus, in a study by Ahmadi and colleagues [49], composite chitosan/gelatin membranes were shown to maintain a continuous release of cinnamon in vitro for more than two weeks, which can provide antibacterial and osteogenic effects. Similarly, Zhu and colleagues have reported a sustained release of metformin in vitro for more than three weeks by the chitosan/polycaprolactone/metformin nanofibrous membrane [38]. Another benefit of nanofiber membranes is their ability to enhance the bioactivity of stem cells which could support the use of these biomaterials in the delivery of cellular therapeutics. For instance, Zhu and colleagues fabricated chitosan/polycaprolactone/metformin nanofibrous membrane which supported the adhesion, proliferation, and osteogenic differentiation of BM-MSCs [38]. Similarly, Ye and colleagues also utilized a type of chitosan/polycaprolactone membrane to assess biocompatibility with BM-MSCs [35]. Specifically, strontium and calcium phosphate nanoparticles were added to the chitosan/polycaprolactone nanofiber membrane and the resulting biomaterial was shown to be suitable for the adhesion and division of BM-MSCs. Moreover, the cells were found to form calcified nodules and release vascular endothelial growth factor (VEGF) indicating their osteogenic and angiogenic differentiation. To date, however, there are quite a few studies that have applied such a strategy in vivo. In a study by Su and colleagues [50], two chitosan membranes were chemically modified in different ways to enhance the durability of the nanofiber structure. The resulting membranes were assessed in a rat calvarial bone defect model and were found to promote bone healing. In particular, the histologic analysis at 8 weeks after treatment with the membranes revealed the formation of a new bone, which appeared almost identical to the natural one. In addition, the micro-computed tomography demonstrated a significant increase in bone density for each of the two chemically modified chitosan membranes. Interestingly, when the chitosan membranes were compared to a commercially available collagen membrane widely used for guided tissue and bone regeneration, the former appeared to be superior in mediating the repair of the osseous defects. In another study [51], a collagen-chitosan membrane was developed using electrospinning technology and utilized to improve bone regeneration in vitro and in a rat model of cranial bone injury. The treatment was associated with high proliferation rates of human periodontal ligament cells in the culture, suggesting that the membranes could be employed in the future for the delivery of cells. The in vivo results of the study also supported the therapeutic potential of the collagen-chitosan membrane. In particular, 8 weeks after the application of the membrane to the bone defect, almost complete healing of the osseous tissue was observed. The newly formed bone was detected by micro-computed tomography and histologic staining. Moreover, both the early marker of osteogenesis, bone alkaline phosphatase, and the late marker of the process, namely, osteocalcin were found to be elevated in the treatment group confirming the active bone formation.

Overall, scaffold, injectable hydrogels, and nanofiber formulations of chitosan hold great therapeutic potential since they have been shown to improve bone repair and regeneration in animal models. It is important to note, however, that the beneficial effects of these biomaterials are mainly due to their composite nature as well as the bioactive molecules and stem cells they deliver.

### 2.2. Cartilage Regeneration Using Chitosan-Based Biomaterials

Articular cartilage, covering synovial joints, is apt for injuries, which if left unhealed, can lead to premature early arthritis and a lower quality of life [52]. Cartilage has a minimal capability for self-regeneration due to its avascular and aneural structure and the lack of mitotic activity of its highly specialized cells—chondrocytes [53]. On top of that, the common clinical treatments of articular cartilage injuries, including autologous chondrocyte implantation and microfractures, do not ensure hyaline cartilage formation, integration of repair tissue into native tissue, nor the complete filling of the injury site [54]. Therefore, treating articular cartilage via regenerative medicine and tissue engineering is of great interest.

Much research is being done to study alternative methods of cartilage engineering using biocompatible and degradable scaffolds that would guide chondrocytes or mesenchymal stem cells (MSCs) to the injury site and promote their further growth and differentiation [53]. Based on the studies, chitosan-based materials are one of the best options for cartilage engineering as they possess inherent antimicrobial and biodegradable properties [55]. Moreover, the N-acetylglucosamine groups of chitosan are structurally akin to glycosaminoglycans present in the extracellular matrix of cartilage tissue, which is attributable to the improved chondrogenesis on the chitosan-derived scaffolds [56].

Hydrogel forms of chitosan-based materials have great potential for clinical research of articular cartilage regeneration, given their high water content and consequent ability to fill in the injury of any shape [57]. Moreover, composite hydrogels can be manipulated to have suitable mechanical and structural properties to further enhance cell proliferation and differentiation. Yang and colleagues conducted a study of 1.5% glycol chitosan/4% dibenzaldehyde-terminated polyethylene glycol hydrogel used for the delivery of adipose-derived MSCs, which showed that the microtubule structure of the hydrogel-facilitated supply of oxygen and nutrients, providing a suitable microenvironment for cell proliferation [57]. Aiming for improved mechanical properties, injectability, and cytocompatibility, Boyer and their team combined silanized hydroxypropymethyl cellulose with silanized chitosan to produce composite hydrogel, which proved to maintain human adipose-derived stem cells viability and secretory activity when transplanted into nude mice subcutis [58]. Further, the injection of the cryogel into a canine model resulted in osteochondral regeneration both in the absence and presence of adipose-derived stem cells, meaning that this hydrogel composition can enhance the repair of osteochondral injuries on its own [58]. Guo and colleagues designed a hydrogel scaffold that mimics microstructures within subchondral lamellar bone via the condensation of cross-linking between chitosan and nontoxic citric acid and ensuing microchannels formation by lyophilization [59]. This novel hydrogel structure supported infiltration, adhesion, proliferation, migration, and chondrogenic differentiation of BM-MSCs in vitro [59]. Rajagopal and colleagues determined that rabbit BM-MSCs deposited more glycosaminoglycans and DNA on multi-layered chitosan-gelatin scaffolds than on randomly aligned ones, amounting to thicker cartilage formation [60]. Nevertheless, the total collagen production was comparable on both chitosan-gelatin scaffolds and consisted mainly of type 2 collagen and lacked type 10 collagen, indicating the hyaline phenotype of regenerated cartilage tissue [60].

Biphasic chitosan scaffolds also show considerable potential for implications in osteochondral tissue engineering by providing the opportunity to compose different microenvironments on different levels [61]. Utilizing this feature of biphasic scaffolds, Nazhvani and their team designed a chitosan bilayered scaffold that mimicked natural cartilage and bone microenvironments (implementing cellulose nanofibers and hydroxyapatite, respectively) for improved integration of regenerated cartilage and subchondral bone. Hypoxic preconditioned buccal fat pad stem cells seeded into this bilayered chitosan scaffold reinforced collagen type 2 and proteoglycans expression, implying hyaline cartilage regeneration [61]. Luo and colleagues further investigated the properties of analogous bilayered chitosan scaffold with a dense cartilage layer and a porous bone layer containing hydroxyapatite [62]. In their study, the research team integrated magnesium and copper ions into the cartilage and bone layers of the scaffold, respectively, to manipulate the mechanical properties of the scaffold and to regulate the biological activity of the seeded cells: Mg^2+^ ions promoted cartilage regeneration, whereas Cu^2+^ ions promoted bone regeneration via upregulated VEGF expression. On top of that, the formation of subchondral bone in the bone layer of the scaffold not only provided a stable mechanical basis for cartilage regeneration but also enhanced the upward movement of newly produced cartilage toward the synovial joint [62].

The biological properties of chitosan-integrated scaffolds can also be implemented to enhance cartilage regeneration. Research work by Li and colleagues [63] demonstrated that synovial MSCs embedded into chitosan hydrogel/3D-printed poly(ε-caprolactone) hybrid scaffolds with recruited tetrahedral framework nucleic acid have great in vivo regeneration potential. As discovered by the researchers, the positively-charged polysaccharides in the chitosan composition can recruit negatively-charged nucleic acid, which enhances the production of cartilage ECM components, differentiation and proliferation of synovial MSCs, and consequently, the fabrication of a microenvironment suitable for advanced cartilage regeneration in vivo [63].

Furthermore, chitosan can be utilized as a part of multilayer composite scaffolds for continuous therapeutics release. In their study, Baharlou Houreh and colleagues demonstrated that multilayer chitosan scaffolds with polycaprolactone mats within and with conjugated kartogenin promoted the chondrogenesis of MSCs [64]. More research by Yuan and their team indicated that chitosan/mesoporous silica nanoparticle microspheres with platelet-derived growth factor-BB and kartogenin-enhanced cartilage regeneration [65]. In their study, Cui and colleagues fabricated a sustained release platform of anhydroicaritin from chitosan hydrogel and mesoporous SiO_2_ nanoparticles, which would lead to chondrogenesis of articular cartilage stem cells, promotion of ECM formation, and consequent in vivo cartilage regeneration [66]. Zhang and colleagues investigated the efficiency of transforming growth factor-β1 (TGF-β1) alone or in cross-linked thiolated chitosan and carboxymethyl cellulose hydrogel [67]. As a result of their study, it was reported that TGF-β1-loaded hydrogels had a higher curative performance as cells within regenerated cartilage tissue and acquired uniform morphology and the ability to produce ECM components and were distributed throughout the scaffold [67]. Singh and colleagues created a hybrid scaffold by implanting platelet-rich plasma/sodium alginate hydrogel into the 3D chitosan/chondroitin sulfate/silk fibroin scaffold that would mimic natural cartilage ECM. The histological analysis of rabbit knee models revealed that the hybrid scaffold has excellent potential for hyaline cartilage regeneration with even cell distribution and high native cartilage ECM depositions, which could lead to better integration of the hybrid matrix into native cartilage at the injury site [68].

Nevertheless, as established by Stager and colleagues, alginate-chitosan polyelectrolyte complex hydrogels, despite the generation of some cartilage at the growth plate injury site, did not produce a significant amount of regenerative response on their own [69]. Still, polyelectrolyte complex hydrogels were able to somewhat reduce “bony bar” formation over physical injury during the first two weeks in another research, entailing the need to use additional therapeutics or growth factors for better tissue regeneration [70]. This implies that chitosan-based scaffolds on their own are a great choice for articular cartilage regeneration but not for the regeneration of other morphologically similar cartilage tissues.

To sum up, chitosan-based hydrogels and scaffolds present a good opportunity for the engineering of articular cartilage, given their effectiveness in different compositions in rat and rabbit models. Nonetheless, more research must be done to assess whether chitosan-based scaffolds can ensure complete hyaline cartilage regeneration in articular cartilage injuries and whether the regenerated cartilage would hold the same mechanical and biological properties as the native cartilage.

### 2.3. Dental Regeneration Using Chitosan-Based Biomaterials

Studies conducted in the field of dentistry have demonstrated the applicability of chitosan in the development of biocompatible dental materials with antibacterial activity. Given the regenerative effects of chitosan on various tissues, additional studies have been initiated to determine its actions on the dental repair. The obtained results indicate the effectiveness of chitosan in aiding the regeneration of various parts of the tooth [71]. In this regard, this subsection will discuss the use of chitosan-based materials for the regeneration of basic dental tissues, including pulp, dentin, and enamel.

Dental pulp (DP) is a connective tissue of neuroectodermal origin, located in the center of the tooth. The main function of this structure is to nourish the dentin, as well as to innervate and protect the tooth [72]. In the case of necrosis in response to noxious stimuli such as caries or trauma, this anatomical structure may lose its ability to self-regenerate [73], further causing complications involving pain and infection to surrounding tissues including the jaw bone [74]. Several studies have shown that experimental chitosan-based biomaterials are able to promote the enhanced preservation and neoformation of DP tissue, therefore suggesting potential usage of the polymer for restoration and conservation of pulp structure.

An essential feature of chitosan is its immunomodulatory property. As previously shown, it has an anti-inflammatory function that allows it to regulate the release of inflammatory factors. In the field of dentistry, one major problem is the health of the oral cavity, which is usually affected by infections and swelling. According to a study by Zhu and colleagues, chitosan can prevent some of these undesirable events, in particular, reduce the inflammatory response and manage the local swelling participating in the degeneration of DP [75]. In the study, they compared a standard pulp capping material, mineral trioxide aggregate (MTA), with injectable bioactive silver-doped glass nanoparticles (Ag-BG) blended with chitosan//β-sodium glycerophosphate gel (CS). Four treatment arms were generated (MTA, Ag-BG, CS, Ag-BG/CS) and the analysis of their cytokine profiles showed that rats treated with Ag-BG/CS had a significant decrease in the levels of inflammatory biomarkers (IL-1β, IL-6 and TNF-α). The data also demonstrated that Ag-BG and chitosan alone were able to suppress the expression of pro-inflammatory cytokines, however, the effect was stronger in combination. This, in turn, suggests that both Ag-BG and chitosan contribute to the anti-inflammatory activity of the hydrogel, providing additional evidence of the modulatory role of chitosan. With regard to pulp preservation, a study in a rat pulpitis model showed that the anti-inflammatory nature of the formulation also allowed DP to preserve its usual condition without signs of degeneration [73].

Another study focused on the use of chitosan-enriched fibrin hydrogel, and investigated its antibacterial role in the regeneration of functional DP. Given that fibrin forms a fibrous network important for hemostasis and subsequent wound healing, this study aimed to test various fibrin-chitosan formulations, containing chitosan as a bacteriostatic component. Using an agar plate diffusion analysis, it was found that the growth of *E. fæcalis* bacteria was reduced by fibrin-chitosan gel treatment, while hydrogel-free controls and fibrin-only hydrogels were less effective. Thus, chitosan was proposed as a powerful component to prevent the invasion of the fibrin scaffold by endodontic residual bacteria, which can disrupt the regenerative process of DP. Moreover, additional experiments were carried out by the group to study the viability and ability of DP-MSCs to release collagen I and III (major components of DP ECM) in hydrogels. It was demonstrated that the addition of chitosan to a fibrin-based scaffold did not alter DPMSC survival, spreading, proliferation, and collagenous matrix production [76], which further suggests its excellent biocompatibility and non-toxic quality.

Similar research conducted by Moreira and colleagues also showed that chitosan in combination with stem cells derived from the apical papilla and photobiomodulation therapy is effective for DP regeneration [73]. As indicated by the results of an in vitro experiment, chitosan in contact with dental stem cells was associated with improved viability, adhesion, and odontogenic differentiation of stem cells originating from the apical papilla. Furthermore, the hydrogel characteristics presented interconnected pores, which facilitated the nutrition of stem cells inside the pores, indicating of the overall stability of the scaffold. This is another property of the polymer that makes it a good candidate for creating porous materials, which are important in terms of their permeability and ability to incorporate and trap viable cells and other compounds. As for the in vivo part of the research, it was shown that the hybrid scaffold (blood clot and chitosan) used in combination with photobiomodulation therapy, formed a well-developed pulp-like tissue in the presence of predentin along the walls of the root canal. This tissue was rich in newly formed vessels with rounded endothelial cell walls, and neither inflammation nor internal/external resorption was identified [73]. Therefore, overall, the results of this study showed that the injectable chitosan hydrogel can promote the three-dimensional spatial organization of endogenous stem cells during the regeneration of the DP without interfering with the positive effect of photobiomodulation therapy.

The next component to be discussed is dentin, which is a yellowish, mineralized avascular tissue that supports the enamel and encloses the central pulp chamber [77]. Dentin has a background similar to that of pulp because both are derived from the neuro-mesenchyme of the dental papilla [78]. Therefore, damage to one structure most often entails damage to another. Several studies conducted in this context have demonstrated the possibility of using chitosan in the restoration of dentin integrity. In most cases, chitosan serves as a platform that releases the active ingredient, and also contains stem cells involved in the remineralization process.

Soares and colleagues in their research on the simvastatin-releasing dental pulp cells (DPC)-chitosan scaffold, explored its potential to deliver bioactive concentrations of simvastatin (0.1 mmol/L) as well as DPCs at the site of interest. As indicated by the results, simvastatin could greatly increase alkaline phosphatase activity and induce mineralized matrix deposition and DPC migration. The beneficial aspects of having a porous chitosan matrix ensure that the migrating and proliferating ability of DPCs remain undisturbed, which once again confirms the fact that the implementation of chitosan has high biocompatibility [79].

Later, the same team conducted a different study with slight modifications to see if it was possible to obtain a cell-free scaffold capable of inducing dentin regeneration with resident cells. While the bioactive component remained the same, chitosan was used as a metal binding agent for the sustained release of Ca^2+^ ions. As it was previously identified, amino groups (-NH2) and hydroxyl groups (-OH) in the composition of the polymer are involved in the process of metal-chitosan interaction; therefore, they were expected to play the role of effective centers for complexing with Ca^2+^. According to the results of a scanning electron microscopy, scientists successfully achieved the formation of a calcium-linked chitosan scaffold, with a constant release of Ca^2+^ and simvastatin. As for its regenerative potential, increased expression of odontoblastic markers was observed in the presence of SIM and calcium hydroxide. They played a synergistic role in DPC differentiation, leading them to deposit the highest amount of the calcium-rich matrix on the scaffold structure and surrounding dentin [80], signifying the promising use of the aforementioned chitosan scaffold as a cell-homing platform for mineralized tissue regeneration.

With a similar approach, Bordini and colleagues studied the applicability of chitosan in combination with calcium aluminate microparticles and 1α,25-dihydroxyvitamin D3 (1α,25VD) [81]. According to the authors, human DPCs in an osteogenic medium containing a calcium aluminate-chitosan scaffold showed an increased proliferation rate, along with an increase in ALP activity which was responsible for mineralized matrix deposition. It has been proposed that the mechanisms underlying the human DPC behavior mediated by the scaffold may be associated with the release of extracellular Ca^2+^ in the medium, with consequent influx to the intracellular environment. Extracellular Ca^2+^ is considered the primary messenger for the regulation of cellular events, including cell migration, cell viability, differentiation, and mineralization [81]. In view of this, calcium-releasing materials such as chitosan are widely used as pulp-capping agents to cause the deposition of a mineralized barrier to seal pulp exposures as calcium levels regulate dentin.

In addition to the functions discussed earlier, Ziotti and his colleagues found a slightly different application for chitosan. They devoted their work to the use of chitosan as a biomodification of demineralized dentin to improve the adhesive interface needed for the formation of the bond between the composite material and dentin [82]. Achieving an adhesive interface is important because carious dentin usually has a disorganized matrix and a heterogeneous morphological structure, which adversely affects degradation time by causing fiber collapse, exposing dentin collagen, and disrupting resin monomer infiltration. In the experiment, scientists induced caries using pH cycling which was then removed and filled with restorative agent resin. A total of 2.5% of chitosan was incorporated into demineralized dentin to investigate its effect on mechanical strength and degradation time of the adhesive interface. The results of the study indicated that chitosan treatment was associated with higher bond strength, indicating its ability to stabilize a hybrid layer. It has been suggested that chitosan cross-links with dental collagen which leads to the formation of a mechanically strong fibrillar chain and thus explains the higher mechanical performance of the chitosan-treated groups in comparison to controls [82].

Having discussed the use of chitosan-based biomaterials in the context of DP and dentin, the remainder of this subsection will focus on chitosan usage for enamel regeneration. Dental enamel is the hardest substance in the human body derived from epithelium that functions as a wear-resistant outer layer of the dental crown. It forms an insulating barrier that protects the tooth from physical, chemical, and thermal damages [83]. Due to the fact that ameloblasts, or enamel-forming cells, gradually disappear as the development is completed, this structure cannot be repaired or remodeled when any defects are observed. Therefore, studies of various approaches to the restoration and protection of this layer are being conducted. For instance, Zhang and colleagues in their work focused on the effect of chitosan pre-treatment in combination with remineralizing agents on artificial enamel white spot lesions [84]. According to the results, the pre-treatment solution did not alter the surface of the white spot lesions, however, when compared with the control group, it could prevent the formation of deep erosions. The assessment of the mechanical properties of the lesions using a Knoop microhardness test also showed that chitosan groups, chitosan-bioactive glass, and chitosan-bioactive glass-polyacrylic acid, exhibited higher hardness values. As proposed by scientists, this could be due to the fact that chitosan captured phosphate ions from the bioactive glass slurry using an electrostatic interaction formed with its NH_3_^+^ groups. On the same basis, it was further assumed that chitosan enhances the delivery of Ca^2+^ due to the interaction with the carboxyl group of polyacrylic acid, which, in the absence of chitosan, chelates Ca^2+^ released from bioactive glass [84]. Importantly, this demonstrates the limited ability of chitosan in the remineralization process; however, the inhibition of ion removal in the case of enamel demineralization is one of its properties that makes it an object of interest today.

The next research group focused on developing a multifunctional system for biomimetic remineralization of acid-etched human enamel. In their strategy, they evaluated a combination of chitosan and agarose in a biopolymer-based hydrogel [85]. Interestingly, the results of the study showed that all grown hydroxyapatite layers of artificial enamel in hydrogels made of agarose only or made of agarose and chitosan had increased microhardness values compared to those etched with acid. However, the values for the chitosan-agarose series were closer to the theoretical value of the carbonated hydroxyapatite of natural enamel. This further proves that CS can act as a bioactive macromolecule in the biomimetic reconstruction of enamel hierarchical structure, and that chitosan-agarose hydrogel can mimic a protein matrix for cost-effective enamel healing [85].

Another chitosan-based hydrogel was developed by Mohabatpour and colleagues as a cell carrier for in vitro regeneration of tooth enamel [86]. The biomaterial complex of oxidized alginate and carboxymethyl chitosan was tested in 3 different compositions (4:1, 3:1 and 2:1 ratios). As shown by live/dead cell analysis, hydrogels provided a favorable three-dimensional environment for the survival of dental epithelial cells (HAT-7) in the absence of the cytotoxic effect. In all groups, the expression of alkaline phosphatase, a marker of enamel matrix mineralization, was found, which indicated the differentiation of HAT-7 cells in ameloblasts and possible restoration of enamel. In addition, hydrogels exhibited antibacterial properties against two cariogenic bacteria: *Streptococcus mutans* and *Streptococcus sobrinus*, which are involved in the formation of dental caries [86]. Taken together, the above findings support the idea of the multifunctional nature of chitosan. In addition to its previously described strengths, the role of chitosan in HAT-7 differentiation can also be distinguished. However, more research is needed to elucidate the mechanisms by which chitosan enhances the differentiation potential of dental epithelial cells.

In conclusion, chitosan is a very versatile natural biomaterial that has been extensively studied in the context of dentistry. It has numerous beneficial properties that allow it, for example, to form electrochemical interactions at the cellular and molecular levels, to inhibit bacterial growth, and to be biocompatible, making it harmless when used in combination with living cells. The purpose of this section was to highlight and discuss the development of new chitosan-based biomaterials as well as the use of chitosan as an important additive for modifying and improving existing dental materials. The application of chitosan biomaterials for the regeneration of bone, cartilage, and dental tissues is summarized in Table 1.

## 3. Skin

Being one of the largest organs in the body, skin serves as a natural barrier against various pathogens and dangers from the external environment. As a consequence, persistent contact with outer factors leads to skin lesions such as wounds, burns, and ulcers [87]. Cutaneous wound healing is a complex process that consists of four overlapping phases, namely hemostasis, inflammation, proliferation, and remodeling. It involves numerous immune and structural cells that secrete signaling molecules, such as cytokines, chemokines, and growth factors, to regulate the healing process. Impairment at any step in this process leads to chronic wound healing characterized by continuous inflammation, persistent infections, and necrosis [88]. The healing of chronic wounds is a long and hard process that is frequently associated with a high percentage of patients’ morbidity and mortality.

**Figure 2:**
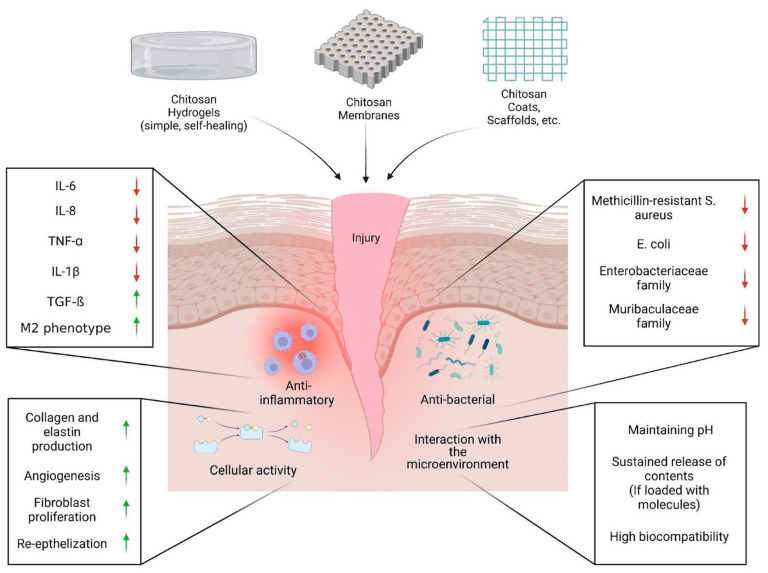
The effect of applying different chitosan biomaterial forms on skin injury. Various forms of chitosan-based materials are studied for the treatment of skin lesions, including hydrogels, membranes, topical gels, sponges, etc. Due to the physico-chemical properties of chitosan those biomaterials work at all stages of wound healing, enhancing skin repair, decreasing fibrosis and promoting anti-inflammatory, antibacterial, and cell proliferative effects.

Drug-delivery systems could provide antioxidative and antimicrobial treatment as well as reduce fibrosis, which in turn, enhances the wound-healing process [89]. Chitosan has been extensively studied in the field of wound healing to produce various wound dressings such as hydrogels, membranes, scaffolds, and sponges (Figure 2). This broad interest is due to their physico-chemical properties including biodegradability, biocompatibility, non-toxicity, and antimicrobial activity [90]. Chitosan-based systems are also hemostatic materials, where molecular weight and deacetylation degree define their effect on blood clotting [4]. In general, chitosan itself plays a role in the first three stages of wound healing. During hemostasis, chitosan facilitates the accumulation of platelets and red blood cells in the wound area and suppresses fibrin dilution leading to reduced bleeding and clot formation. In the next two stages, chitosan promotes antibacterial protection and induces granulation tissue formation that accelerates tissue proliferation [31]. However, using chitosan alone for skin regeneration is limited due to its poor mechanical properties. Those limitations can be avoided by adding additional materials such as synthetic polymers which helps to improve the strength and elasticity of chitosan-based drug delivery systems [91]. Moreover, loading chitosan-based materials with antibacterial, hemostatic, and immunomodulatory drugs and bioactive compounds may further enhance its positive effect [92,93]. Overall, chitosan-based drug-delivery systems are now considered good wound dressing materials to be potentially used for healthcare purposes.

### 3.1. Skin Tissue Regeneration and Wound Healing Using Chitosan-Based Hydrogels

Chitosan-containing hydrogels are extensively used in various wound healing studies and are already integrated into clinical practice. High interest and demand for this material is caused by such features of chitosan-based hydrogels as biocompatibility, biodegradability, and its ability to carry several types of bioactive molecules such as drugs, antibiotics, etc. Moreover, a chitosan hydrogel shows its beneficial effect on cutaneous wound healing owing to its resemblance of tissue structures, control of loaded substances release, and so on. Some studies have shown that modified hydrogels have the capacity to induce self-healing of wounds alone, even when no additional substances are added.

A peculiarity of a good wound-healing biomaterial is its positive interaction with an environment. The hydrogels based on chitosan derivatives greatly enhance wound healing by providing a moist environment and cell proliferative assistance at the wound site. These properties of the chitosan hydrogels allow them to reduce wound area by 100%, while other alternatives, regular gauze dressings achieve only 72.9% in wound area reduction [94]. Chitosan hydrogels are also capable of adhering to the wound surface firmly through Schiff base and amide reactions, facilitating fast hemostasis. For instance, the whole-blood-clotting assay conducted in vitro by Xie and colleagues has shown great adherence of red blood cells to the carboxymethyl chitosan/oxidized dextran/sodium alginate hydrogel, indicative of good hemostasis at the site of tail amputation [95]. Moreover, the cross-linked structure of the hydrogel resembles the 3D structure of the extracellular matrix and promotes wound healing. Li and colleagues have demonstrated that complete wound healing of a full-thickness skin defect model and epithelial reconstruction can be achieved within 14 days post-treatment when using chitosan-poly (ethylene glycol)-dihydrocaffeic acid [96].

Chitosan-based hydrogels show good biocompatibility and hemocompatibility when tested in vitro. Thus, in their study, Yang and colleagues obtained chitosan-based hydrogel wound dressing via photopolymerization. A newly developed hydrogel has shown great biocompatibility, with a hemolysis percentage of 1.97–2.79% [97]. In addition, chitosan-based biomaterials show immunomodulatory functions by stimulating the activation of epithelial and immune cells. The stimulation of wound healing is seen from the production of proliferative cellular signals, such as interleukins and growth factors, and the activation of corresponding cells. A recent study showed that after sulfation, the chitosan derivative not only retained its biocompatibility but also acquired increased anticoagulant activity and the potential to bind IL-6 and TGF-β. Apart from its proinflammatory activity, IL-6 stimulates megakaryopoiesis and participates in inducing the differentiation of human myeloid cell lines, while TGF-ß exerts chemotactic and pro-mitotic activities essential for the function of granulation tissue during the proliferative phase of wound healing [98,99]. Pro-healing M2 phenotype of macrophages plays a key role in inhibiting inflammation caused by an immune response and re-epithelization of the wound area by producing a variety of growth factors [88]. Sulfated chitosan facilitated the differentiation of macrophages towards the M2 phenotype, increasing their number to almost 50% compared to regular chitosan. Throughout 30 days post-treatment, the sulfated chitosan showed better re-epithelization and vascularization than a regular chitosan hydrogel [100]. Positive interactions with host connective tissue often lead to a higher metabolic activity of the cells and upregulated production of extracellular matrix components. Andrade del Olmo and colleagues formulated chitosan-PEG hydrogel by cross-linking chitosan with polyethylene glycol diacid, which markedly increased metabolic activity to almost 95% as well as the amount of collagen and elastin produced in ulcerated wounds [101]. In vivo studies on diabetic and nondiabetic rats conducted by Sheir and colleagues showed enhanced closure of pressure ulcers, increased collagen production, and higher maturation of granulation tissue [102].

Chitosan-based hydrogels also show anti-inflammatory and antibacterial effects. Various cytokines and molecules are involved in the inflammatory response, including but not restricted to TNF and IL-1 family cytokines [103]. Chitosan derivatives can effectively interact with cellular signaling pathways and the compounds mentioned above, which are involved in the immune system’s inflammatory response to skin damage. For instance, the study by Hao and colleagues revealed that a chitosan/sodium alginate/velvet antler blood peptides hydrogel reduced acute inflammatory response mainly via the activation of PI3K/AKT/mTOR/HIF-1α/VEGFA pathway and suppression of TNF-α and IL-1β expression [104]. Both of these factors play an essential role in activating immune response and initiation of inflammation. For instance, both TNF-α and IL-1β participate in the initiation of the formation of the inflammasome during an immune response [103]. Moreover, chitosan can affect the expression of various cell surface markers. During its activity, the chitosan hydrogels can greatly decrease markers expressed mainly by macrophages and monocytes, such as CD68, during the inflammatory phase of the immune response and increase the expression of the endothelial cell surface markers such as CD31, indicative of increased angiogenesis [97,104]. Aside from its hemostatic properties, the chitosan hydrogel demonstrated antibacterial activity against Methicillin-resistant Staphylococcus aureus. Therefore, chitosan-based hydrogels can be applied to treat drug-resistant bacteria-infected skin wounds and chronic wound inflammation, making it a promising alternative in treating infected wounds [97].

One feature of chitosan-based hydrogels is their ability to self-heal. The so-called self-healing hydrogels can be best characterized when referring to their ability to return to their primary shape after any mechanical manipulations. This characteristic is caused by various ionic and hydrogen bonds prevailing in those types of hydrogels. Guo and colleagues, for instance, combined quaternary ammonium chitosan with tannic acid to give hydrogel the ability to form dynamic ionic bonds, which helped the hydrogel to recover under 350% strain [105]. Moreover, extensive hydrogen bonds can be obtained by cross-linking chitosan and polyethylene glycol diacid [101]. This unique property of the hydrogels to self-heal enables them to return to their original shape after any physical manipulations. This allows its use in healing stretchable wounds on highly mobile organs such as limbs [101].

Self-healing hydrogels show such features as inhibition of inflammation, suppression of bacterial growth, and facilitation of skin proliferation [105]. These properties can be further enhanced by loading hydrogels with bioactive compounds, including antibiotics and exosomes containing other molecules. For example, self-healing chitosan hydrogels loaded with exosomes can induce angiogenesis, fibroblast proliferation, and collagen deposition [106]. Hydrogels loaded with antibacterial antibiotics such as cefuroxime, tetracycline, and amoxicillin show their sustained release, resulting in better antibacterial activity against, for instance, *Staphylococcus aureus* and *Escherichia coli* [101]. In addition, self-healing hydrogels possess immunomodulatory features that are particularly important when it comes to persistent pain caused by the secretion of pro-inflammatory cytokines, which can eventually lead to chronic inflammation. Self-healing hydrogels can downregulate the expression of pro-inflammatory cytokines, such as TNF-ɑ, IL-6, and IL-8, and thus reduce painful inflammation in patients [101]. Finally, biocompatible self-healing hydrogels show effective hemostatic properties, which accelerate wound repair. Self-healing chitosan hydrogel produced by mixing chitosan with a graphene oxide showed adhesiveness and hemocompatibility in vitro. In vivo studies on rat liver bleeding and full-thickness skin defect models demonstrated remarkable hemostatic effects and accelerated wound healing [107].

The chitosan hydrogels do not simply satisfy expectations regarding wound healing but, in some cases, even outperform other alternatives. The chitosan hydrogels, for instance, polyethylene glycol monomethyl ether modified glycidyl methacrylate functionalized chitosan and N-succinyl chitosan-oxidized hyaluronic acid-based hydrogel, show outstanding performance compared to the commercially available alternatives such as Tegaderm™ Film and Arista™, respectively [97,108].

Thus, chitosan-based hydrogels can acquire a variety of properties, such as increased wound healing, accompanied by anti-inflammatory and antibacterial properties. Positive interactions with host cells and simultaneous inhibition of infectious bacterial growth are also desirable characteristics of chitosan hydrogels. Modifications of chitosan hydrogels, such as their ability to self-heal, make them suitable for the treatment of a variety of skin injuries. In some cases, chitosan hydrogels may even outperform the commercially available analogs prescribed for the treatment of damaged skin.

### 3.2. Skin Tissue Regeneration and Wound Healing Using Chitosan-Based Membranes

Some types of skin lesions require temporary barriers that support a moist environment, gaseous exchange, and cell proliferation as well as possess antimicrobial properties and drug release [90]. Chronic wound healing requires materials that tightly adhere to wounds, while absorbing exudate and supporting the reduction in the fibrotic area [109]. Ideally, a good wound dressing should work at all stages of wound healing, facilitating fast hemostasis, clearance from bacterial invasion, production of granular tissue, and wound scarring. Those functions can be found in membranes. They have similar effects on wound healing as hydrogels. In addition, chitosan-based membranes effectively mimic natural ECM due to the similarity of chitosan with the glycosaminoglycans that are found in ECM [110]. However, studies emphasize the effect of chitosan membranes on their antibacterial properties and ability to interact with the host cells effectively due to their poor electrospinnability and physico-chemical properties. This causes some limitations to the use of chitosan-based membranes in biomedical applications.

Doan and colleagues reported that 95% of the wound area healed after 10 days post-treatment with the multilayer membrane [111]. The newly grown skin shared a resemblance with the native tissue and illustrated the progressive construction of vascular systems, hair follicles, etc. In addition, nanofibers formed from polyurethane blended with modified chitosan were loaded with linezolid to enhance the wound-healing process in diabetic rats. Linezolid incorporated into nanofibers stimulated more effective wound healing and promoted an antibacterial effect [112].

Chitosan-based membranes and scaffolds are known for their antibacterial properties. A multilayer membrane for facilitating wound healing, which consists of oligomer chitosan, polycaprolactone, and polyvinylpyrrolidone, was assembled by Doan and colleagues [111]. According to the results, the composite membrane was able to inhibit the growth of several bacteria types. An antibacterial effect showed chitosan/polyethylene oxide nanofibers armed with antibacterial silver and zinc oxide nanoparticles. The scaffold showed antibacterial effects against *Staphylococcus aureus*, *Escherichia coli*, and *Pseudomonas aeruginosa* [113]. Similar results were reported by Cui and colleagues, who reported the antibacterial properties of membranes loaded with corresponding chemicals [114].

The chitosan membranes were also reported to interact with host cells, especially with fibroblasts, which are essential for wound healing and the formation of connective tissue. Bagheri and colleagues demonstrated that the chitosan nanofibers facilitated fibroblast migration and proliferation. In their research, Cui and colleagues prepared a specialized chitosan/polycaprolactone membrane and achieved the controlled release of ciprofloxacin, as well as stimulated the activation and accumulation of fibroblasts, in the injured area [114]. Moreover, the membrane loaded with 2% ciprofloxacin illustrated the best biocompatibility, which is another property of chitosan membranes. Another experiment conducted by Zhou and colleagues involved a bilayer membrane consisting of chitosan and poly(ε-caprolactone). In addition to the properties recognized in previously discussed studies, the research revealed that the membrane was non-cytotoxic to fibroblasts when loaded with 1.2% ZnO. Additionally, the bilayer membrane alleviated inflammation and stimulated re-epithelization when tested in vivo on mice [115]. A similar nanofiber membrane formed via the electrospun method from polycaprolactone and chitosan oligosaccharides was loaded with quercetin and Rutin which are known for their good effects in wound healing. Chitosan oligosaccharides improved the hydrophilicity and water absorption of the scaffold. Overall, the nanofiber showed antibacterial and antioxidant properties in the treatment of burn injuries [116]. The nanofiber formulated from electrospun poly(vinyl alcohol)/chitosan-g-poly (N-vinyl imidazole) with incorporated titanium dioxide/curcumin was tested on wound-healing properties and showed full skin regeneration in 14 days, as well as an antibacterial effect against *Escherichia coli* and *Staphylococcus aureus*, where 90% of bacteria stopped their growth within 1 h after application [117].

The effectiveness of chitosan-based membranes in cutaneous wound healing is greatly enhanced when they are loaded with various bioactive compounds. Membranes support a controlled and sustained release of active components. According to the study results, chitosan-polycaprolactone fibrous membrane could sustain the release of ciprofloxacin into the wound for 15 days [114]. Chitosan/polyvinyl alcohol membranes were loaded with sodium nitroprusside doped prussian blue nanoparticles and collagen I by using an electrospinning method. Due to the properties of chitosan, the nanofibers mimicked natural ECM, increased antibacterial effect, and enhanced fibroblasts proliferation, which eventually resulted in accelerated wound healing [118]. Another study generated a new nanofiber formed from chitosan and polyethylene oxide with the addition of various active components, including manuka honey, propolis, insulin, and L-arginine. The scaffold showed a positive effect for the treatment of chronic wounds due to its antimicrobial and antioxidant properties, in particular against *Staphylococcus aureus* strain [110]. Lastly, the newly formed nanofiber for diabetic ulcer treatment was formed from chitosan and polyvinyl alcohol with the incorporation of ursolic acid. The membrane mimics natural ECM, as well as has surface hydrophilicity and wettability and controlled release of ursolic acid. Moreover, it possesses anti-inflammatory properties by suppressing M1 and stimulating M2 phenotype of macrophages, inhibiting TNF-α and IL-6 expression, and reducing ROS production. The in vivo study revealed an improved closure rate and enhanced revascularization and re-epithelization [119].

Chitosan-based membranes can improve wound healing and accelerate tissue regeneration due to their hemocompatible, immunomodulating, and antibacterial properties. Moreover, they stimulate fibroblast migration and accumulation in the injured sites. Finally, membranes support a moist environment, gas exchange, and exudate elimination, which make them a great tool for the treatment of chronic wounds.

### 3.3. Skin Tissue Regeneration and Wound Healing Using Other Chitosan-Based Biomaterials

Apart from hydrogels and membranes, there are also other forms of chitosan-derived biomaterials that facilitate wound healing and skin regeneration. For example, coats, sponges, topical gels, and chitosan dressings are used in tissue engineering skin regeneration studies. These forms of chitosan biomaterials possess the same properties as hydrogels and membranes. However, they lack some physical features such as an ability to self-heal or to support the sustained release of agents. Some skin injuries require a more practical application of the chitosan, which can be achieved by developing its different physical forms.

In the study of Wang and colleagues, chitosan coats’ effectiveness, biocompatibility, and antimicrobial activity in surgical wounds were evaluated and compared with regular gauze dressing [120]. The comparative analysis of the cytolytic activity of WS1 fibroblasts incubated with either negative control or chitosan dressing did not show a significant difference, indicating its great biocompatibility. The chitosan dressing inhibited the growth of infection-causing biofilm-forming bacteria, including Enterobacteriaceae (8.1% to 7% in 6d post-surgery) and Muribaculaceae (3.3% to 2.3% in 6d post-surgery). Another infection-causing bacteria Enterococcaceae, which is the major cause of postoperative wound infection making up most of the post-surgery bacterial population, illustrated lower abundance in chitosan dressing (25.2%) compared to regular gauze (32.2%) on day 6 after surgery. According to the authors, the excellent antimicrobial activity of chitosan coats is the result of the presence of numerous amino acids being positively charged at acidic pH, which contributes to the effective lysis of bacterial cells. The antimicrobial activity of chitosan was shown in a study by Chong and colleagues where they investigated the wound-healing activity of chitosan particles after a loop electrosurgical excision procedure [121]. Antibacterial properties were caused by the chitosan’s free amine groups binding to the bacterial cell wall. In their work, Wu and colleagues studied both the in vitro and in vivo activity of chitosan-modified bioactive glass paste. Accordingly, chitosan neutralized the pH, sustaining the bioactivity of bioactive glass. The in vivo antibacterial activity and proliferative activity of bioactive glass enhanced upon modification with chitosan and outperformed the activity of both biomaterials pastes when used separately.

Another study showing the efficacy of the chitosan particles for the treatment of skin lesions is a randomized placebo-controlled clinical trial of the topical gel containing chitosan, chlorhexidine, allantoin, and dexpanthenol [122]. Wound healing was considered ‘good’ in 22.2% of the placebo group and 97.3% of the experimental group 7 days after the surgical removal of the lower third molars, while at the period of 2 days after surgery, only 41.7% and 0% of the wound healing activity were considered “good” in the placebo group and treatment group, respectively. Sáez-Alcaide and colleagues suggest that the results were achieved because of the antimicrobial activity of chitosan against Gram-positive and Gram-negative bacteria and fungi as well as chlorhexidine particles’ preventive properties against alveolar osteitis.

The study conducted by Sultana and colleagues confirms the results discussed earlier. A freeze-dried sponge with an antibacterial component was formed via mixing tempo-oxidized nanocellulose and chitosan, where chitosan controlled the pore size and nanocellulose was responsible for the mechanical stability of biomaterial. The new scaffold showed adaptivity to the wound environment by degrading and releasing antibacterial components at a pH lower than neutral. In vivo studies on rat tail amputation and skin-wound models also displayed hemostasis and outstanding wound closure [123].

Currently, chitosan-based hydrogels and membranes are the most extensively used forms of biomaterials for the treatment of skin lesions such as wounds, burns, and ulcers. They enhance cutaneous wound healing as well as promote immunomodulatory, antibacterial, and local cell proliferative activity, activate cellular metabolism, and reduce scar size. The ability to be loaded with additional bioactive compounds pushes the borders of their versatility even further. The moist environment, gaseous exchange, and exudate absorbance found in chitosan-based membranes make them an excellent option for the treatment of chronic wounds. Additionally, some chitosan modifications can highly enhance its physico-chemical properties and generate such forms as multilayer membranes and self-healing hydrogels for the healing of cutaneous wounds susceptible to high physical activity and strain. Finally, there are also other forms of chitosan for wound healing, such as sponges, topical gels, and coats. Depending on the complexity of the skin injury, chitosan can possess many forms with a variety of functions, which makes it a great tool when dealing with skin injuries.

## 4. Cardiac and Nervous Tissues

### 4.1. Cardiac Tissue Regeneration Using Chitosan-Based Biomaterials

Over the years, several types of treatments have been developed for cardiovascular diseases, which are one of the leading causes of death worldwide. For many decades conventional treatment modalities were used to treat cardiovascular diseases, such as surgical interventions, implantation of a pacemaker, administration of pharmaceutical drugs, and in extreme cases, heart transplantation [124]. However, the main drawback of these types of treatment is that they mainly focus on reducing symptoms and overlook the regeneration of lost cardiac tissue in the region of injury [125]. Many studies have been conducted to find a biomaterial suitable for cardiac tissue engineering, and chitosan has proven to be one of the polymers that can be successfully used in cardiac tissue regeneration procedures. The main reason is that chitosan possesses properties similar to glycosaminoglycans that are widely distributed throughout the connective tissue, making it an ideal candidate for tissue repair [126]. Different types of chitosan have been used in cardiac tissue regeneration, specifically scaffolds, injectable hydrogels, and stem cell delivery using chitosan hydrogels as a platform [127].

The application of chitosan scaffolds is a promising therapeutic approach in cardiac tissue regeneration as they possess the necessary features of a biomaterial such as biocompatibility and biodegradability. The main two effective ways of fabricating chitosan scaffold are electrospinning and 3D-printing [128,129]. However, during the development of biomaterial for cardiac tissue regeneration, it is essential to create not only a biocompatible and biodegradable scaffold but also a biomaterial with high electrical conductivity since the cardiac muscle is an electrically active tissue responsible for the transmission of electrical signals through the heart and with strong cell adhesion properties to reflect the ECM of cardiac tissue [130]. In order to meet the desired properties for proper tissue regeneration, the usage of more than one polymer is required. Hence, composite materials were added to a scaffold to enhance and comply with the necessary properties. Chitosan scaffolds can exhibit needed properties; however, they have limitations because of their weak electrical conductivity and high rate of degradation. Therefore, recent studies focused on finding proper chitosan composite scaffolds in order to improve their electrical and biological properties for better cardiac tissue regeneration. In the in vitro study by Jiang and colleagues [30], graphene oxide was added to the chitosan scaffold to improve its electrical properties and to better mimic the characteristics of the native myocardium [30]. It was found that adding graphene oxide into the chitosan scaffolds increases the electrical conductivity of graphene oxide establishing the signal transduction between the cells and the scaffolds, resulting in increased expression of the connexin-43 protein, which is an important myogenic signal transmitted by cardiomyocytes [30]. Specifically, chitosan/carbon scaffolds were proven effective in enhancing electrical properties in a rat myocardium model [131]. The chitosan/carbon nanotube/polyvinyl alcohol was tested in vivo in this model and was proven effective in transmitting signals and regenerating damaged cardiac tissues due to unpaired carbon nanotube electrons that help in better cell attachment promoting conductivity [132].

The usage of chitosan alone is not applicable in the proliferation of cells due to a positively charged polysaccharide, resulting in poor cell adhesion. Therefore, cardiomyocytes cannot attach and survive on pure chitosan scaffolds. Tamimi and colleagues suggested adding alginate to the chitosan scaffolds as a negatively charged polysaccharide that binds with chitosan and forms a more robust chitosan biomaterial. Mainly, porosity and pore size allow cell adhesions to form because they are essential in facilitating the penetration of nutrients and migration of cells throughout the whole surface of the scaffold. The MTS assay was used to evaluate the in vitro compatibility of the constructed scaffolds by monitoring the survival and proliferation of MSCs. In an ideal situation, the porosity of the scaffold should be equal to or more than 90% to permit penetration. In the study, the level of achieved porosity was 96%, which is an ideal condition for the uptake of nutrients on the surface of the scaffold [133]. Furthermore, Ahmadi and colleagues also reported that a polyurethane/chitosan/carbon nanotube nanofibrous scaffold could enhance chitosan’s proliferation properties due to increased surface roughness and protein absorption [134].

Chitosan hydrogels are a type of chitosan formulation known as highly biocompatible in cardiac tissue regeneration. Hydrogels are effective because they are water-insoluble, crosslinked polymer matrices with high water content (more than 30%) and it is easy to inject them into damaged heart tissues, which can regenerate cardiac tissues with higher effectiveness [135]. Chitosan hydrogels are proven to be more effective in usage with other composites [136]. Moreover, combinations of natural polymers such as chitosan and synthetic polymers can prevent burst degradation of the hydrogel, maintaining its mechanical integrity in compliance with native cardiac tissue [137]. In a study by Torabi and colleagues, chitosan hydrogel containing gold nanoparticles and poly glycerol sebacate was created with high electrical conductivity, porosity, and temperature-sensitive properties for better resemblance to cardiac tissue. As a result, the created chitosan hydrogel supported cell attachment and growth. The chitosan hydrogel demonstrated the necessary electrical conductivity for the cardiac tissue, hence, can be used as a cardiac tissue regeneration biomaterial [138]. Another derivative of gold polymer considered to be effective in cardiac tissue engineering is gold nanoparticles (AuNP) distributed throughout the chitosan hydrogel. It was found that the concentration of AuNP can significantly influence conductive properties and cell viability [139]. Mokhtari and colleagues (2020) also utilized chitosan composite hydrogels loaded with AuNP and concluded that it was a practical approach to improve degradation resistance and mechanical strength because all the components of hybrid collagen can be mixed by an intermacromolecular Schiff base crosslinking reaction [140]. Furthermore, a higher concentration of AuNP in chitosan hydrogel resulted in higher expression of connexin-43 protein and improved cardiomyocyte morphology [141]. In addition to chitosan hydrogels with AuNP, another hybrid hydrogel containing poly-pyrrole-chitosan has also demonstrated its effectiveness in cardiac tissue regeneration. The composite hydrogel was injected into a rat myocardial infarction model after the 7th-day post-MI. The microelectrode arrays analysis showed that injection of the hydrogel increased field potential amplitude and faster conduction velocity in the damaged myocardium. In addition, ECG analysis showed hydrogel shortened prolonged QRS/QT intervals induced by the MI and led to cardiac function improvement [142]. Using the same rat MI model, Domenge and colleagues injected acellular chitosan hydrogel and measured the impact of the degree of acetylation of the chitosan on cardiac tissue. The authors concluded that the 24% degree of acetylation improved the functionality of damaged myocardium due to a significant decrease in fibrosis and hypertrophic stress. The chitosan hydrogel provided structural support for the damaged ventricle and generated a repair mechanism for cardiac tissue [28].

In the clinical treatment of acute MI, stem cell transplantation has not become widespread due to the low survival rate of transplanted cells. Therefore, it was suggested that stem cells should be delivered using a platform. In a study by Yao and colleagues, it was proven that the utilization of chitosan bioactive hydrogel as a platform for the delivery of stem cells maintains the cells’ survival rate. The effectiveness of the chitosan hydrogel was measured by the survival of human placenta-derived mesenchymal stem cells (hP-MSCs), which were transplanted with chitosan hydrogel into a mouse MI model. The fate of hP-MSCs was monitored by utilizing the green fluorescent protein assay using firefly luciferase in vivo in mice. Data from the assay demonstrated that chitosan hydrogels could provide a suitable microenvironment for the survival of hP-MSCs. Moreover, the survival of stem cells enhanced the regeneration of the cardiac tissue by expressing proangiogenic cytokines after transplantation [143]. Another approach to increasing the survival rate of mesenchymal cells with the help of chitosan was suggested by Liu and colleagues [29]. The authors co-transplanted chitosan hydrogel with BM-MSCs in vivo into a mouse model with MI. Effective delivery of stem cells proved to restrict inflammation by producing anti-inflammatory cytokines and reducing myocardial damage. The study showed that the delivery of MSCs with chitosan improved their survival rate, minimized inflammation and regenerated cardiac tissue [29]. Another study performed by Liu and colleagues (2021) also concluded that bone-marrow-derived MSCs survival and recovery of cardiac tissue were improved by using chitosan hydrogel that protected the vascular endothelial cells from pyroptosis and inhibited the inflammatory response [144]. Moreover, nanodots containing hMSCs and encapsulated into chitosan/collagen hydrogel matrices improved the angiogenesis of the heart, leading to the enhanced functionality of the left ventricle ejection fraction [145].

To sum up, different types of chitosan, such as scaffolds, injectable hydrogels, and stem cells delivered by hydrogels, have proven to be effective as therapeutic treatments for cardiac tissue engineering. Nevertheless, it should be noted that the chitosan biomaterial’s effectiveness was mainly enhanced by adding different composites that improved the biomaterial’s biological properties, such as electrical conductivity, mechanical strength, cell adhesion, and porosity.

### 4.2. Nervous Tissue Regeneration Using Chitosan-Based Biomaterials

For many years, the lack of functional treatments and the disadvantages of existing therapies have encouraged the search for new therapeutic approaches to counteract CNS and PNS injuries [146]. In this context, chitosan is an attractive material because of its mechanical strength, porosity, biodegradability, and biocompatibility. It has also been used for repairing nerve injury, either alone or in combination with other biomaterials as well as with adhesion molecules, cells, or growth factors [147]. Moreover, one of the potential mechanisms of chitosan to accelerate the healing rate of the nervous system is enhanced adhesion of neuronal cells on its membrane. It was also revealed that chitosan fibers can demonstrate a superior attachment and migration of Schwann cells which reconstructed axons to the Bungner bands in the nervous system [25]. However, properties of natural polymers such as a lack of sufficient mechanical strength and fast degradation can affect the effectiveness of the treatment. A potential strategy to address these challenges is combining synthetic and natural polymers. As a result, synthetic polymers can provide mechanical strength and long-term degradation, maintaining cell attachment sites and the cytocompatibility properties of natural polymers such as chitosan [148]. Nowadays, popular chitosan-based biomaterials used for nervous tissue regeneration are mostly in the form of scaffolds and hydrogels.

Chitosan has been widely used for the fabrication of different scaffolds in nerve tissue regeneration. Chitosan scaffolds can be placed in the nerve channels as a guiding substitute in order to promote regeneration both in the central and peripheral nervous systems. Recently, several research groups have developed new chitosan scaffolds in combination with other polymers for nerve tissue regeneration. Pooshidani and colleagues fabricated a conductive scaffold using chitosan in combination with polycaprolactone for nerve tissue engineering. The addition of gold nanoparticles and sacrificial fiber (40% of polyethylene oxide) helped to achieve a desirable interconnected porosity, formed a spherical shape of gold nanoparticles with narrow particle size distribution (126 ± 20 nm), and improved surface hydrophilicity (75–80%) of the scaffold. The results of FE-SEM and MTT assay showed that the scaffold had no cytotoxic effect on stem cells and can support spindle-shaped morphology similar to mature Schwann cell cultures with some proliferation of cells [149]. Another research group demonstrated the fabrication of poly (L-lactic acid) (PLLA)/chitosan-based scaffold by using a liquid–liquid phase separation technique. Blending chitosan with PLLA (3 *w*/*v*%) helped to achieve better structural properties such as desirable porosity, improved hydrophilicity, slowed degradation rate, and pH alteration. On MTT assay, the human neuroblastoma cells attachment on the scaffolds increased from 78.2% in pure PLLA to 94.3% in PLLA/chitosan 3 *w*/*v*%, while DPPH assay revealed a significant decrease in cell viability on PLLA scaffold compared to PLLA/chitosan scaffold. Moreover, pure PLLA demonstrated more cell death rate and blending with chitosan diminished the cytotoxicity compared to the pure PLLA scaffold [150]. In another study, three chitosan-based composite scaffolds were prepared by using chitosan/hyaluronic acid (HA)/gelatin (Gel), chitosan/collagen (Col), and chitosan/polyethylene glycol (PEG). The addition of other materials to chitosan reduced the average pore size (chitosan/HA/Gel (114.57 ± 11.36 μm), chitosan/Col (109.93 ± 14.48 μm), and chitosan/PEG (93.93 ± 10.32 μm)), while improving the mechanical properties of the composite scaffold in comparison with pure chitosan. Furthermore, the evaluation of these composite scaffolds for biocompatibility and rat pheochromocytoma (PC12) cell neuron growth demonstrated that Cs/PEG scaffold possessed a higher cell survival rate without cytotoxicity compared to other scaffolds with the promotion of adhesion, proliferation, and differentiation of the cells [151]. A recent in vivo study also demonstrated that chitosan scaffolds can be effectively used to repair central and peripheral nerve tissue injury. Si and colleagues (2019) also prepared chitosan/collagen composite scaffolds for peripheral nerve regeneration. Compared to a collagen-only scaffold, the combination with chitosan reduced the pore size of the scaffold, its liquid uptake and degradation rate, and increased the mechanical property of the chitosan/collagen composite scaffolds. The chitosan/collagen composite scaffolds also demonstrated good cytocompatibility without cytotoxicity on L929 fibroblasts, RSC96 cell lines, and primary Schwann cells with particular facilitation of the attachment, migration, and proliferation of Schwann cells. Furthermore, in vivo subcutaneous implantation on rabbits demonstrated that the addition of chitosan to a collagen scaffold can show a modulated degradation behavior without an inflammatory reaction [152]. Another experiment on rats showed that a graphene oxide-composited chitosan scaffold contributes to the functional recovery of the injured spinal cord. The prepared chitosan–graphene oxide scaffold with an 18–87 μm porous structure was transplanted into a T9 total resected rat spinal cord. The results demonstrated nerve cell growth into the pores between chitosan molecular chains, promoting angiogenesis, neuron migration, and neural tissue regeneration, thus initiating the repair of damaged special cord nerve tissue. Further analysis of behavioral and electrophysiological results suggests that the chitosan–graphene oxide scaffold has the potential to significantly restore the locomotion function and somatosensory evoked potentials of rats [153]. In a study by Liu and colleagues, chitosan scaffolds were loaded with nerve growth factors to repair long-segment (20 mm) sciatic nerve defects in adult rats. The results showed that their chitosan scaffold can maintain a release of nerve growth factor for 8 weeks and implantation of the scaffold to a 20 mm gap significantly promoted the recovery of motor and sensory functions 12 weeks after surgery. Moreover, the treated sciatic nerve was reconnected with neurons successfully establishing functional neural circuits similar to normal sciatic nerves. In addition, the sciatic nerve also promoted reconnection of the motor endplate with the target muscle restoring its contractive function [154].

Hydrogels containing chitosan have also been shown to promote nerve tissue regeneration. Particularly, there are several recent in vitro and in vivo studies reporting the use of material with chitosan in order to enhance the repair of CNS injury. For example, a semi-interpenetrating polymer network injectable hydrogel with polymer network and linear polymer were developed with the incorporation of HA into the chitosan-based self-healing hydrogel for CNS regeneration. The addition of HA allowed the hydrogel to pass through a 26 gauge needle easily, while in vitro, the spreading, migration, proliferation, and differentiation of incorporated neural stem cells were increased due to the unique structure of semi-interpenetrating polymer network hydrogel. Moreover, in vivo experiments on zebrafish and rat models of CNS injury and intracerebral hemorrhage, respectively, demonstrated better biocompatibility of the gel, CNS injury repair, and functional recovery in the groups treated with the aforementioned hydrogel compared to pure chitosan self-healing hydrogel [155]. Revkova and colleagues (2020) also used a chitosan hydrogel to demonstrate its ability for neural stem cell differentiation. H9-derived neural stem cells and directly reprogrammed neural precursor cells were seeded on the films from chitosan-g-oligo(L,L-lactide) copolymer hydrogel and demonstrated enhanced adherence and signs of spontaneous differentiation along the neuronal lineage, with no cytotoxicity. In addition, the biomechanical properties of the hydrogel were comparable to that of a human spinal cord with a dissected pia mater [156]. Moreover, the combination of chitosan-based hydrogels with stem cells can effectively promote the regeneration of CNS injury. A recent study on the animal model of spinal cord injury demonstrated that implantation of biomimetic composite hydrogel of chitosan- and gelatin-containing endometrial stem cells can promote the growth of neuronal cells and recover the sensory and motor functions of adult rats during the 6 weeks after injury. Additional injection of atorvastatin during the treatment is necessary in order to attenuate neuroinflammation, and increase locomotion performance and recovery of the hind limb functions, demonstrating that hydrogel can be used in combination with other treatments [157]. Furthermore, chitosan-based hydrogels can also be applied similarly for peripheral nerve regeneration. Liu and colleagues recently developed a new tubular chitosan-based nerve-guide hydrogel conduit (CNHC) with increased mechanical flexibility. The results of mechanical tests suggest that the hydrogel implant exhibits satisfactory strength and flexibility and is suitable for connecting the nerve ends in its wet state. CNHC also promoted C6 cell proliferation and adhesion and further analysis on a rat model of sciatic nerve injury demonstrated that implantation of the CNHC has comparable functional recovery to autograph nerves 90 days after surgery. Histological analysis also indicated that the CNHC promoted the growth of axons and SCs and guided axons through the conduit to reach a distal stump bridging nerve ends [158].

Furthermore, the addition of factors and stem cells can promote the regenerative effects of the chitosan hydrogel in peripheral nerve injury. A recent study on a rat model of long-distance sciatic nerve defects showed that aligned chitosan nanofiber hydrogel grafted with peptides mimicking bioactive brain-derived neurotrophic factor and vascular endothelial growth factor can repair a 15 mm nerve defect, facilitating nerve regeneration, vascular penetration, and functional recovery. The hydrogel also promoted the proliferation of SCs through the PI3K/AKT signaling pathway, guiding the regeneration of axons [159]. Xu and colleagues (2021) also fabricated poly(D, l-lactic acid) (PDLLA)/β-tricalcium phosphate (β-TCP) nerve conduits filled with injectable chitosan-HA hydrogels for repairing injured peripheral nerves. They demonstrated that chitosan-HA hydrogels loaded with nerve growth factors are suitable for the adhesion, spreading, and differentiation of neuronal cells, promoting a sustained release of the factors for over 6 weeks. Further in vivo experiments on rats suggested that PDLLA/β-TCP nerve conduits laden with chitosan-HA/nerve growth factor hydrogels have the potential to repair 10 mm sciatic nerve defects with a significant positive effect on axon regeneration and myelination [160]. The combination of chitosan with alginate was used for the transplantation of olfactory ectomesenchymal stem cells (OE-MSCs) to promote peripheral nerve regeneration. The results of the MTT and LDH assay showed that hydrogel is cytocompatible with OE-MSCs and can sustain cell viability. The aforementioned hydrogel made of chitosan and alginate and loaded with OE-MSC hydrogel was further injected into a 3 mm sciatic nerve defect of Wistar rats, enhancing nerve regeneration and functional recovery compared to the control group and hydrogel without cells 8 weeks after the procedure [161]. The same Alg/chit hydrogel was used for the delivery of 4-Methylcatechol, which is known for its ability to stimulate the synthesis of nerve growth factor and brain-derived neurotrophic factor to promote nerve regeneration. 4-MC loaded into the hydrogels showed no cytotoxic effects and improved proliferation of PC12 cells. The results of functional and histopathological tests demonstrated that 10% of 4-MC loaded into the hydrogel significantly enhanced sciatic nerve regeneration after 8 weeks compared to the negative group [162], thus concluding that fabricated hydrogel can also be used as a suitable delivery material not only for stem cells but also for other compounds.

Overall, recent studies demonstrate the efficacy of chitosan-based scaffolds and hydrogels for the treatment of CNS and PNS injuries. Moreover, the beneficial effects of these chitosan-based materials can be enhanced by their composite nature and bioactive molecules and stem cells incorporated into them. Therefore, chitosan has great therapeutic potential for the regeneration of nerve tissues. The use of chitosan-based biomaterials for cardiac and nervous tissue regeneration is summarized in Table 2.

## 5. Conclusions and Future Perspectives

Chitosan-based materials can acquire a variety of properties, such as increased regeneration and healing, accompanied by anti-inflammatory and antibacterial properties. It can also initiate positive interactions with host cells and simultaneous inhibition of infectious bacterial growth. The analysis of recent experimental studies demonstrated that chitosan is a promising therapeutic biomaterial for bone, cartilage, dental, skin, nervous, and cardiac tissue regeneration. However, as a natural polymer, chitosan lacks sufficient mechanical strength and electrical conductivity and has a high degradation rate. In order to overcome these obstacles, chitosan was combined with different composites, mostly with synthetic polymers, which improved its biomechanical properties such as electrical conductivity, mechanical strength, porosity, long-term degradation of the material, and cell adhesion while maintaining cell attachment sites and cytocompatibility. Modification of chitosan-based materials in the form of hydrogels, scaffolds, nanoparticles, and membranes allows them to be suitable for a variety of treatment methods including implantation and injection. Moreover, the incorporation of growth factors, stem cells, and therapeutic agents can further improve their regenerative/healing effects on bone, skin, and cardiac and nervous systems.

Chitosan-based materials have huge commercialization potential in various biomedical fields. Chitosan is a cheap material and can be obtained from various sources such as crustacean shells, insect cuticles, and fungal cell walls. Animal-derived chitosan can be purchased for 10 to 1000 US dollars per kg, while the estimated cost for fungal chitosan is from 50 to 5000 US dollars per kg [163]. It has practical applications in different industries such as agriculture, pharmacy, textile, and cosmetology. In the last decade, chitosan also received much attention in the biomedicine and food industry [164]. It is approved by FDA as a safe food additive and there are several nutritional supplements available for use in chronic kidney disease in pets, as a safe weight loss supplement and cholesterol-reducing and antioxidant agents [165]. The recent analysis of clinical trials on biomaterial and therapeutic applications of chitosan demonstrates that more than 100 trials are underway to investigate the safety and efficacy of chitosan [166]. However, despite its wide application, chitosan currently has been approved by the FDA only for very few biomedical applications such as wound-dressing material and nerve conduit [167,168]. There is also a chitosan scaffold for cartilage repair approved for clinical use in Europe and Canada [169,170]. The main regulatory challenges of chitosan for clinical approval are its source of extraction, purity, and characterization. Chitosan may have impurities such as high bio-burden, heavy metals (lead and mercury), and bacterial and protein contamination. The total protein content and tropomyosin in animal-derived chitosan can cause hypersensitivity to the material leading to the development of an allergy. The degree of deacetylation, varying from 70% to 90%, may also affect the chemical behavior and bioadhesion of chitosan [166]. These issues can be resolved partially by switching to a fungi-derived chitosan. Fungal chitosan is free of allergenic animal source protein, heavy metals, and the degree of deacetylation and molecular weight can be controlled during the production process [163]. Therefore, for further approval in the biomedical field by European and US regulatory agencies, chitosan may be required to obtain a thorough material characterization, a safety profile of the end product, and a warranty of product consistency from manufacturers and commercial suppliers. Successful clinical translation of chitosan-based materials and their marketing depends on how clinical trials, followed by manufacturers and suppliers, can resolve issues such as the toxicity and safety challenges, development of standardization techniques, and understanding the potential long-term adverse effects. With the advent of new technologies and methods as described in this review, these current limitations of chitosan could be eliminated and new clinically approved chitosan-based biomaterials can be seen in the near future.

## Figures and Tables

**Table 1:** Use of different chitosan formulations for bone, cartilage, and dental tissue regeneration.

Tissue	Formulation	Model	Effects	Reference
Bone	Composite scaffold of chitosan and magnesium oxide nanoparticle-coated eggshell particles loaded with BMP2	Rat model of calvarial bone defects	Enhanced new osseous tissue formation, increased bone defect closure	[39]
	Composite biomimetic scaffolds made of chitosan and gelatin and loaded with dental pulp cells	Mouse model of immunodeficiency	Increased mineralization, enhanced formation of the new bone	[40]
	Composite scaffolds made of chitosan and gelatin	Mouse model of femur orthotopic implantation	Enhanced formation of new extracellular matrix	[41]
	Injectable hydrogel made of glycol chitosan and oxidized hyaluronic acid and loaded with graphene oxide	Rat model of calvarial bone defects	Enhanced closure of bone defects	[42]
	Thermosensitive hydrogel/nanoparticle system made of chitosan and glycerol phosphate and loaded with vancomycin	Rabbit model of chronic osteomyelitis	Reduced bone inflammation, enhanced bone repair	[43]
	In situ forming hydrogel consisting of methacrylated glycol chitosan and montmorillonite	Mouse model of calvarial bone defects	Increased new osteoid bone formation	[45]
	Electrospun nanofiber membranes made of Triethylamine/tert-butyloxycarbonyl or butyryl-anhydride modified chitosan	Rat model of calvarial bone defects	Enhanced formation of new bone which appeared almost identical to a natural one	[50]
	Electrospun nanofiber membrane made of collagen and chitosan	Rat model of cranial bone injury	Enhanced healing of the osseous tissue	[51]
Cartilage	1.5% Ethylene glycol chitosan/4% Dibenzaldehyde-functionalized-polyethylene glycol hydrogel	Rat model of knee joint articular cartilage injury	Improved cell proliferation, thicker layer of regenerated tissue that fused well with adjacent cartilage, differentiation of stem cells into neonatal chondrocytes similar in morphology to hyaline chondrocytes	[57]
	Multilayer scaffold of chitosan hydrogel and polycaprolactone mat conjugated with kartogenin	Human adipose-derived stem cells	Chondrogenic differentiation of SCs, increased expression of SOX9, COLL2, and ACAN	[64]
	Silanised hydroxypropymethyl cellulose and silanised chitosan hydrogel	Canine model of osteochondral defect	Improved osteochondral regeneration in load-bearing defects	[58]
	Chitosan-based hydrogel and mesoporous SiO_2_ nanoparticles loaded with anhydroicaritin	Rabbit model of cylindrical cartilage defect in trochlear groove	Increased extracellular matrix production, improved cartilage regeneration	[66]
	Multi-layered chitosan-gelatin scaffold	Rabbit model of bilateral osteochondral defects	Improved hyaline cartilage regeneration	[60]
	Chitosan hydrogel/3D-printed poly (ε-caprolactone) hybrid that recruited tetrahedral framework nucleic acid	Rabbit model of knee defects	Improved cartilage regeneration, impeded the development of osteoarthritis	[63]
	Alginate-chitosan hydrogels	Rat model of physeal injury	Decreased bony bar formation, increased chondrogenic differentiation in fast-degrading scaffold, increased bony bar formation in slow-degrading scaffold	[70]
	Chitosan/mesoporous silica nanoparticles microspheres loaded with kartogenin and platelet-derived growth factor BB	Rabbit model of focal cartilage defects	Improved chondrogenic differentiation in vitro, improved cartilage regeneration in vivo	[65]
	Chitosan, polyvinyl alcohol, and citric acid hydrogel scaffold	Rat model of osteochondral defects in femoral groove	High biocompatibility of the scaffold that mimicked subchondral lamellar bone structure, almost complete in situ cartilage regeneration	[59]
	Cross-linked thiolated chitosan and carboxymethyl cellulose hydrogel loaded with TGF-β1	Rat model of full-thickness cartilage defects in knees	Regenerated cartilage tissue, homogeneous cell morphology, even cell distribution	[67]
	Platelet-rich plasma and sodium alginate-based hydrogel embedded in the porous 3D chitosan, chondroitin sulfate, and silk fibroin scaffold	Rabbit model of full-thickness articular cartilage defect	Increased hyaline cartilage ECM deposition, improved integration of regenerated tissue with native cartilage	[68]
	γ-Poly- glutamic acid, carboxymethyl chitosan, and bacterial cellulose bilayer scaffold with a dense cartilage layer containing Mg^2+^ and a porous osteogenic layer containing nano-hydroxyapatite and Cu^2+^	Rabbit model of osteochondral defects in knee joints	Improved cartilage and subchondral bone regeneration	[62]
	Bilayer chitosan scaffold with cellulose nanoparticles in cartilage-facing layer and hydroxyapatite in bone-facing layer	Rabbit model of articular cartilage defects in trochlear groove	Improved cartilage regeneration, improved subchondral bone integrity	[61]
	Alginate-chitosan polyelectrolyte complex (PEC) hydrogel	Rat model of growth plate injury	Improved cartilage regeneration, not impeded bony bar formation	[69]
Dental	Simvastatin (SV)–releasing chitosan-calcium-hydroxide (CH-Ca) scaffold	Rat model of calvarial defects	Improved mineralization in vivo	[80]
	Injectable chitosan hydrogel scaffold	Rodent model of orthotopic dental pulp regeneration	Enhanced dental pulp regeneration	[73]
	Injectable oxidized alginate-carboxymethyl-chitosan hydrogel	Rat incisor HAT-7 dental epithelial cell line	Increased HAT-7 cell survival and differentiation potential	[86]
	2.5% Chitosan solution	Human sound molar teeth	Improved bond strength in demineralized dentin	[82]

**Table 2:** Use of Different Chitosan Formulations for Cardiac and Nervous Tissue Regeneration.

Tissue	Formulation	Model	Effects	Reference
Cardiac	Chitosan with graphene oxide scaffold	Heart H9C2 cells	Desirable porosity, improved electrical conductivity and cell viability, increased cell adhesion, enhanced expression of specific cardiac proteins (connexin-43)	[30]
	Chitosan/carbon nanotube/polyvinyl alcohol scaffold	Rat mesenchymal stem cells	Achieved desirable porosity, improved electrical conductivity, increased cell proliferation and adhesion	[132]
	Chitosan with alginate scaffold	Human mesenchymal stem cells	Achieved desirable porosity, increased cell attachment and decreased cell viability	[133]
	Chitosan/polyurethane/CNT nanofibrous scaffold	Cardiac rat myoblast cells	Improved electrical conductivity, increased surface roughness and cell proliferation	[134]
	Injectable chitosan/pluronic/gold-decorated cellulose nanofiber hydrogel	Cardiac rat myoblast cells	Achieved desirable porosity, increased cell adhesion and proliferation, slow degradation	[135]
	Pluronic/chitosan hydrogel containing gold nanoparticles and poly glycerol sebacate	Cardiac rat myoblast cells	Achieved desirable porosity, improved electrical conductivity, increased cell adhesion	[138]
	Gold nanoparticles distributed throughout the chitosan hydrogel	Rat mesenchymal stem cells	Achieved desirable porosity, improved electrical conductivity and cell viability, increased cell adhesion	[139]
	Chitosan/collagen injectable hydrogel containing gold nanoparticles	Mouse fibroblast cells	Achieved desirable porosity, improved electrical conductivity, increased mechanical strength	[140]
	Poly-pyrrole-chitosan hydrogel	Rat myocardial infarction model	Reduced fibrotic scar resistivity and enhanced electrical conduction	[142]
	Acellular chitosan hydrogels	Rat myocardial infarction model	Increased repair mechanism, permanent coronary ligation in rats	[28]
	Chitosan hydrogel with Immobilized insulin-like growth factor-1 and incorporated with human placenta–derived mesenchymal stem cells	Human placenta-derived mesenchymal stem cells	Increased angiogenesis, improved survival rate of stem cells	[143]
	Chitosan hydrogel with bone marrow-derived mesenchymal stem cells	Mouse model of myocardial infarction	Improved survival rate of stem cells, minimized inflammation, increased regeneration of cardiac tissue	[29]
Nervous	Conductive scaffold of chitosan and polycaprolactone with gold nanoparticles	Schwann cells (SCs) extracted from sciatic nerves of the 2- to 3-day-old Wistar rats	Improved hydrophilicity with desirable porosity and no cytotoxicity, facilitated proliferation of stem cells supporting their spindle-shaped morphology similar to mature Schwann cells	[149]
	Poly (L-lactic acid)/chitosan-based scaffold	Human neuroblastoma cells	Improved hydrophilicity with desirable porosity and slow degradation, increased cell attachment and decreased cell viability	[150]
	Chitosan/polyethylene glycol scaffold	Rat pheochromocytoma (PC12) cells	Improved mechanical properties, no cytotoxicity, increased cell survival rate, adhesion, proliferation and differentiation	[151]
	Chitosan/collagen composite scaffold	L929 fibroblasts, RSC96 cell lines and primary stem cells Subcutaneous implantation on rabbits	Reduced pore size, improved mechanical properties, no cytotoxicity, facilitated attachment, migration, proliferation of stem cells Modulated degradation and no inflammatory reaction after implantation	[152]
	Graphene oxide-composited chitosan scaffold	Rat model of spinal cord injury	Repaired damaged spinal cord nerve tissue, promoted angiogenesis, restored locomotion	[153]
	Chitosan scaffold with nerve growth factor	Rat model of sciatic nerve defect	Reconnected the nerve with neurons, restored motor and sensory functions	[154]
	Hyaluronic acid/chitosan-based self-healing injectable hydrogel	Zebrafish and rat models of CNS injury and intracerebral hemorrhage	Increased biocompatibility, improved functional recovery and CNS repair	[155]
	Biomimetic composite hydrogel of chitosan and gelatin with endometrial stem cells	Rat model of spinal cord injury	Promoted growth of neuronal cells, recovered the sensory and motor functions	[157]
	Tubular chitosan-based nerve-guide hydrogel conduit	Rat model of sciatic nerve injury	Promoted functional recovery, growth of axons and Schwann cells through conduit	[158]
	Aligned chitosan nanofiber hydrogel grafted with peptides mimicking bioactive brain-derived neurotrophic factor and vascular endothelial growth factor	Rat model of long-distance sciatic nerve defects	Repaired 15 mm nerve defect, facilitated nerve regeneration, vascular penetration and functional recovery	[159]
	Injectable chitosan/hyaluronic acid hydrogel with nerve growth factor	Rat model of sciatic nerve defect	Promoted sustained release of factors, repaired nerve defect, improved axon regeneration and myelination	[160]
	Alginate/chitosan hydrogel with olfactory ectomesenchymal stem cells and 4-Methylcatechol	Rat model of sciatic nerve defect	Enhanced nerve regeneration and functional recovery without cytotoxicity	[161,162]
